# Temporal features of sitting, standing and stepping changes in a cluster-randomised controlled trial of a workplace sitting-reduction intervention

**DOI:** 10.1186/s12966-019-0879-1

**Published:** 2019-11-21

**Authors:** Samantha K. Stephens, Elisabeth A. H. Winkler, Elizabeth G. Eakin, Bronwyn K. Clark, Neville Owen, Marj Moodie, Anthony D. La Montagne, David W. Dunstan, Genevieve N. Healy

**Affiliations:** 1 School of Public Health, The University of Queensland, Herston Road, Herston, Brisbane, QLD 4006 Australia; 2 Baker Heart & Diabetes Institute, Melbourne, Australia; 3 Centre for Urban Transitions, Swinburne University of Technology, Melbourne, Australia; 4 Deakin Health Economics, Institute for Health Transformation, Deakin University, Geelong, Australia; 5 Work, Health & Wellbeing Unit, Centre for Population Health Research, Deakin University, Geelong, Australia; 6 Mary MacKillop Institute for Health Research, Australian Catholic University, Brisbane, Australia; 7 School of Sport Science, Exercise & Health, University of Western Australia, Perth, Australia; 8 School of Exercise and Nutrition Sciences, Deakin University, Melbourne, Australia; 9 School of Public Health & Preventive Medicine, Monash University, Melbourne, Australia; 10 School of Physiotherapy, Faculty of Health Sciences, Curtin University, Perth, Australia

**Keywords:** Workplace, Office workers, Sitting time, Sedentary, Intervention, Compositional data analysis

## Abstract

**Background:**

There is now a body of evidence on the effectiveness of interventions to reduce workplace sitting time. However, there has been limited reporting of how such interventions may impact behaviour both during and outside of work. Sitting, standing and stepping changes following a workplace intervention were examined across five timeframes (work time on work days; non-work time on work days; work days; non-work days; overall (i.e. work and non-work time on all days)), and the relationships between changes during and outside of work was assessed.

**Methods:**

The cluster-randomised controlled trial, ‘Stand Up Victoria’, delivered a multi-component workplace-delivered intervention that successfully reduced workplace and overall sitting time (relative to controls). Separately, over the five timeframes, changes in device (activPAL3)-assessed outcomes — sitting; prolonged sitting (≥30 min bouts); standing; and, stepping — were compared between intervention (*n* = 114) and controls (*n* = 84), along with the time-course of sitting changes during work hours, using mixed models. The potential relationships of changes during work with changes outside of work were examined using compositional data analysis.

**Results:**

On workdays, intervention participants significantly (*p* < 0.05) improved their activity profile relative to controls, with reduced sitting (− 117 min/8-h workday, 95% CI: − 141, − 93) and prolonged sitting (− 77 min/8 h workday, 95% CI: − 101, − 52); increased standing (114 min/8 h workday, 95% CI: 92, 136) and maintenance of stepping (3 min/8 h workday, 95% CI: − 7, 11, *p* = 0.576). Effects were nearly identical for time at work; similar but slightly weaker for overall; and, small and non-significant outside of work on workdays and non-work days. Improvements occurred at all times, but not equally, during work hours (*p* < 0.001). Correlations between changes during and outside of work on workdays were very weak in both the intervention group (*r* = − 0.07) and controls (*r* = − 0.09).

**Conclusions:**

Sitting time was reduced almost exclusively during work hours (via replacement with standing), with reductions evident during all working hours, to varying degrees. There was no evidence of compensation, with minimal change in activity outside of work, in response to changes in activity at work. Future interventions may benefit from exploring how best to elicit change throughout the whole day, and across work and non-work domains.

**Trial registration:**

This trial was prospectively registered with the Australian New Zealand Clinical Trials register (ACTRN12611000742976) on 15 July 2011

## Background

The associations of high levels of sitting time with adverse health outcomes (including premature mortality) have been well-reported [1]. To counteract the premature mortality risks associated with too much sitting, adults need to participate in an estimated 60 min or more of daily moderate to vigorous intensity activity [2]. Desk workers accrue much of their daily sitting time in the workplace [3] and, as such, reducing sitting time in this setting has become a priority for both public and occupational health [4]. Reviews have identified several intervention trials that have led to reductions in workplace sitting time, with the greatest changes related to environmentally-focused approaches that include provision of sit-stand workstations, particularly as part of multi-component interventions [5, 6]. Changes have primarily occurred in workplace sitting time or in sitting time overall [5], with some changes identified in patterns of sitting-time accrual, such as the duration and number of sitting bouts [7–9].

By contrast, the extent to which, and manner in which, a workplace-delivered sitting-reduction intervention may impact on activity outside of the work setting is not well understood. It is possible that the interventions may prompt changes in the primary work domain directly and in other domains (e.g. non-work). For example, yielding reductions in sitting during leisure time (generalisation). Another possibility is that of compensatory effects, such that reducing workplace sitting results in increased leisure-time sitting or reduced levels of physical activity [10]. These latter changes would be consistent with the predictions of the ‘ActivityStat’ or ‘EnergyStat’ hypothesis, namely, that increased activity or energy expenditure in one domain triggers compensatory decreases elsewhere [11]. Compensation is sometimes raised as a possible explanation when the overall intervention effect is less pronounced than the effect in the primary domain [11]. In considering compensation and generalisation, rather than only identifying the degree of changes in multiple behaviours and domains, further exploring how they interrelate may yield further insights. This can be achieved using compositional data analysis (CoDA), which allows the consideration of time-use across multiple domains that sum to a fixed total, such as 24 h [12]. An alternative potential explanation for why domain-specific effects are sometimes larger than overall effects is that effects may be confined to the primary domain of intervention (e.g., the workplace) and the degree of overall effect is proportional to the amount of exposure to that domain (e.g., time at the workplace). That is, lack of exposure to the relevant domain dilutes the intervention effects. Understanding the interplay between changes in various domains and behaviours (which broadly may be characterised as generalisation, compensation, and dilution) can inform future research and the consideration of potential future regulations or policies.

Beyond considering sitting, standing and stepping time within domains (e.g., at work; outside of work), investigating the time-course of sitting changes within the primary intervention domain may elicit important information about whether certain times throughout the day may be prone to greater or lesser change. For example, different effects around lunchtime might be suggestive of more discretionary opportunities to not sit, which may have ramifications for postprandial glucose and lipid metabolism [13]. Differences between later versus earlier in the day may reflect behaviour changes relating to discomfort or fatigue [14]. Few studies have examined such change in temporal patterns of sitting time following intervention. One study that examined hourly changes was the Stand Up Comcare trial, the pilot study for the Stand Up Victoria intervention reported here [7]. In the pilot study, reductions in sitting time (relative to controls) were observed at every hour of work time, but not in equal proportions, with mornings showing the largest changes, and some evidence of a diminished effect around the typical lunch period (12–1 pm) [7]. As that pilot trial used a non-randomised design with a small number of participants from one workplace [15], it is important to identify whether these temporal effects are replicated in other studies.

To address these evidence gaps, data were used from Stand Up Victoria [16] — a cluster-randomised controlled trial of a multi-component workplace-delivered intervention aimed primarily at reducing workplace sitting time. The effectiveness of the intervention on sitting and activity outcomes has been reported [9], demonstrating a significant and substantial reduction in total workplace sitting of more than 1.5 h at three-month follow up relative to controls, with sitting replaced primarily with standing, and minimal or no impact to stepping. This present study did not aim to re-evaluate the effectiveness of this intervention, but rather aimed to provide an in-depth examination of when changes did and did not occur, with a view to informing whether there was potential generalisation, compensation and/or dilution. Intervention effects over five timeframes covering work time and non-work time; and temporal variations in effects on workplace sitting (the primary outcome) were examined. The relationships between changes during work and non-work time were also explored using compositional data analysis (CoDA) techniques.

## Methods

### Study design, participants and recruitment

Stand Up Victoria was conducted in Melbourne, Australia from 2012 to 2014. The methods [16], intervention development [17], worksite characteristics [18] and primary activity outcomes [9] have been published elsewhere. The intervention complied with the CONSORT guidelines, a populated checklist CONSORT checklist is provided in Additional file 1. A populated TIDieR checklist for interventions is provided in Additional file 2. In summary, 14 geographically-separate worksites were recruited into the trial from a single organisation and cluster randomised 50:50 to receive the intervention (*n* = 7; with 136 workers) or control (n = 7; with 95 workers). Ethics approval was obtained through the Alfred Health Human Ethics Committee (Melbourne, Australia), with all participants providing written, informed consent. Additional ethics approval was granted by the University of Queensland, School of Public Health Research Ethics Committee (Brisbane, Australia) for these analyses.

### Intervention

The primary aim of the intervention was to reduce workplace sitting time, using intervention elements [16] directed at the individual (e.g., health coaching and motivational interviewing by trained health coaches); the workplace environment (e.g., sit-stand workstations); and, the organisation (e.g., management consultation and emails from worksite managers). An initial face-to-face coaching session included the set-up of the workstation, and was followed by telephone calls in weeks 2, 4, 8 and 12. The intervention messaging focused on three key intervention targets: ‘Stand Up’ at least every 30 min throughout the workday; ‘Sit Less’, reducing the total time spent sitting by replacing it with standing (gradually progressing towards approximately 50% sitting and standing); and, ‘Move More’ by including more incidental movement throughout the work day. The intervention primarily focused on workplace behaviour. However, workers were also encouraged to implement strategies to reduce sitting time outside of the workplace, with the third telephone coaching call (week 8) specifically targeting sitting reduction and increased activity outside of the workplace. The intervention group received feedback on their sitting, standing and stepping time both during work hours and across the whole day (during waking hours) following baseline and three-month assessments. At the end of 3 months, the workstations were retained, however no further intervention was provided. Workers at the control sites were advised to continue usual activities and received written feedback on their baseline and three-month activity outcomes shortly after the three-month follow up.

### Data collection

The study collected data at baseline, three-months (immediately following the intervention period) and 12-months after baseline via: self-administered questionnaires; a face-to-face assessment that collected anthropometric and blood biomarker data; and a seven-day continuous activity monitoring component. Socio-demographic and work-related data were collected at baseline only. Baseline and three-month (intervention period) data only were used for this study.

### Activity outcomes

The activPAL3™ thigh-worn activity monitor (PAL Technologies Limited, Glasgow, UK) provides valid measures of sitting, standing, stepping and postural transitions [19]. Participants were asked to wear the activPAL3™ activity monitor 24 h per day for seven consecutive days at each time point, while recording their sleep/wake and work times (commencement/finish) in a diary. Full details of the protocol and data processing have been reported previously [9]. Briefly, data were processed in SAS 9.4 (SAS Institute Inc., Cary NC, USA). Time spent in the following activities — sitting/lying, referred to as sitting; prolonged sitting in ≥30 min bouts; standing; and, stepping — were extracted for the timeframes relevant to this study. Non-wear time and sleep time were not included. Valid days required wear for ≥80% of work hours, and ≥ 10 waking hours wear time (when waking hours were inferred from movement). The five timeframes of interest and their detailed definitions are provided in supplementary material (Additional file 3: Table S1). Data were reported over standardised timeframes (16 h waking days, 8 h work time, 8 h non-work time, 60 min/hour).

### Covariates

Baseline body mass index (BMI) was assessed as weight (kg)/ height (m^2^), collected objectively as described previously [9]. Self-report questionnaires collected age, sex, and Physical Quality of Life scores (*PhysQoL* AQoL-8D) from the Assessment of Quality of Life survey instrument, with the standard psychometric scoring [20]. Diary data were used to calculate two indicators of each participants’ working hours at baseline: how many days per week they worked, and how long per day they worked. These were calculated for Monday–Friday only, which was the only scheduled work for the study workplace, with weekend work being short periods of additional work, rather than full work days.

### Statistical analyses

**Fig. 1:**
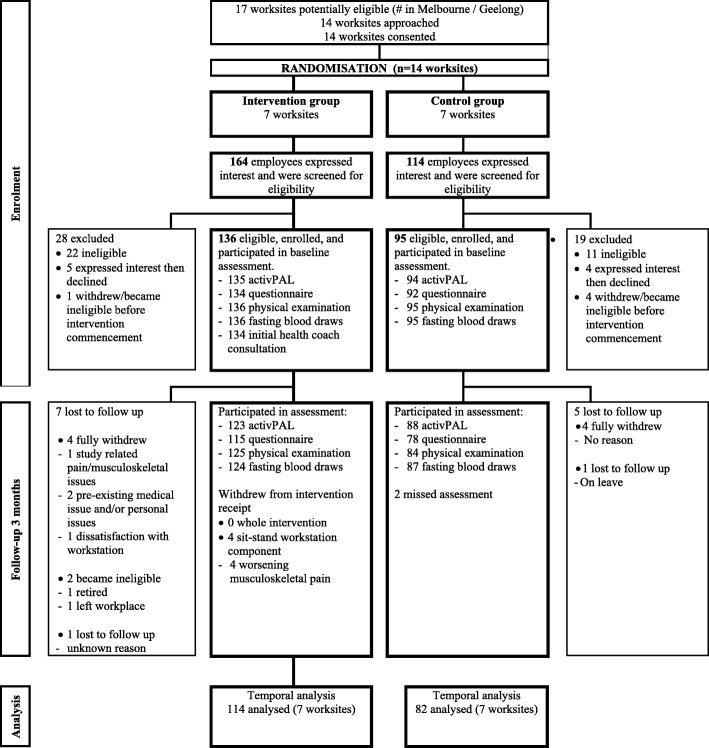
Flow diagram for recruitment, participation and analyses (baseline to 3-month follow up)

Statistical analyses were performed in SPSS Statistics Software version 25 (SPSS, Inc., Chicago IL, USA) unless specified otherwise. Linear mixed models, in STATA version 15 (STATACorp LP) were used to examine the within-group changes and differences between groups in sitting and activity outcomes, adjusting for baseline values of the outcome and potential confounders, correcting for clustering via random intercept. Potential confounders (age, sex, BMI, *PhysQoL* AQoL-8D) were chosen as an a priori list based on findings from the trial’s main outcomes [9], with further consideration of the extent of work occurring (days worked and average daily hours worked on weekdays). For better comparability of effects across timeframes, complete case analysis was used, further limiting analyses to those with the requisite data available for all of the relevant timeframes (Fig. 1).

Mixed models were also used to investigate temporal effects during work hours (the primary domain of intervention). These models accounted for repeated measures (i.e., two time-points, multiple days, multiple hours per day), clustering (random intercept), adjusted for potential confounders (as above) and days of the week, and included the effects of hour, group (intervention/control), and time-point (baseline/3 months) along with their two and three-way interactions. Temporal effects were calculated for both subjective time (i.e., time since starting work) and clock time (i.e., hours of the day). Models were performed in SAS 9.4 (SAS Institute Inc., Cary NC, USA). These models were limited to the participants with data on the covariates, and baseline and 3-month data on work-specific sitting, standing, and stepping time.

#### Relationship between activity during and outside of work hours

Compositional Data Analysis (CoDA) methods were applied, using the packages ‘compositions’ and ‘plyr’ in R software version 3.5.1 (R Foundation for Statistical Computing, Vienna, Austria). Time use on workdays (24 h) at baseline, and at 3 months, was initially divided into seven components: work activity (sitting, standing, stepping); non-work activity (sitting, standing, stepping); and, sleep/non-wear (all remaining time), with the total summing to 24 h. To simplify this, the composition was then recalculated with just the six waking components (i.e., the work and non-work activities). All activity categories within this timeframe were mutually exclusive. Changes (baseline to 3 months) were then calculated using the perturbation method [21]. Participants without valid data on work or non-work hours (on workdays) were excluded.

The relationships between changes in sitting, standing and stepping at work and outside of work were then explored (for the intervention and control groups separately), using correlations and covariance bi-plots. Covariance bi-plots are an exploratory tool that visualises the relationship between the various components (e.g., work sitting and non-work sitting) by displaying them against the two main components extracted from principal components analysis, based on the centred log-ratio (CLR) transformation [22]. The mutual relationship between each pair of components is displayed via their links. Angles indicate the extent and direction of relationship between components (0° = perfect direct relationship; 180° = perfect inverse relationship; 90° = possibly uncorrelated). The overall degree of relationship can be quantified by a correlation coefficient [22, 23]. The interpretation of the correlations and variability in the bi-plots is limited by the reliance on the CLR transformations. Accordingly, further description is provided via log-ratio scatterplots, which do not rely on this transformation.

## Results

### Participant characteristics

The overall characteristics of Stand Up Victoria participants, including their work tasks and the spatial characteristics of their office spaces, have been described in detail elsewhere [9, 18, 24, 25]. Eligible participants for the present study had a mean (± SD) age of 45.9 ± 9.8 years in the control group (*n* = 82), and 44.9 ± 8.9 years in the intervention group (*n* = 114), with 72.8 and 64.9% females respectively. In both groups, most eligible participants reported being employed at 1.0 full-time equivalent capacity (65 controls, 80.2% and 92 intervention, 80.7%). At worksite level, job tasks were phone-based (*n* = 4), non-phone based (*n* = 7) or mixed, with both phone and non-phone based tasks (*n* = 3) [24]. The baseline characteristics of eligible participants were comparable to those excluded for lack of available data (Additional file 1: Table S2). Compliance with the monitoring was stable over time, and not significantly different between the groups, based on the number of valid days and the duration of wear time per day, which consistently averaged just under 7 days and 16 h per day overall (Additional file 3: Table S3). The duration of work versus non-work time on workdays (just over and just under 8 h, respectively) was consistent between groups and over time. There was a slight shift over time in the intervention group, with the number of workdays declining slightly between baseline and 3 months (− 0.21 [95% CI: − 0.42, − 0.01], *p* = 0.044) with a corresponding, but non-significant, increase in the number of non-work days (0.16 [95% CI: − 0.04, 0.36], *p* = 0.115). Overall, approximately 62% of the valid days were workdays (i.e., 4.4 days/week).

### Effects on sitting and activity at work and outside of work

Intervention effects on sitting, standing, and stepping outcomes over each timeframe are shown in Table 1. Relative to controls, those in the intervention group significantly reduced their daily sitting time by 78 min (95% CI: − 98.1, − 58.4) overall (i.e. work and non-work time on all days). These same, but slightly larger effects were seen on work days (− 117.1 min/16 h [95% CI: − 141.0, − 93.2]). Within workdays, effects were only seen during work hours (− 109.5 min/8 h [95% CI: − 130.8, − 88.2]) with small and non-significant effects during non-work hours (− 6.8 min/8 h [95% CI: − 17.0, 3.4]). Effects on sitting time were also very small and non-significant on non-workdays (− 0.7 min/16 h [95% CI: − 29.3, 30.7]). Corresponding effects of a similar magnitude were seen in increased standing time (overall, on workdays and during work time) with no large or significant effects seen at other times. No large or significant effects were seen in stepping in any timeframe (ranging from − 0.5 min/16 h on non-workdays [95% CI: − 13.4, 12.4] to 2.6 min/16 h on workdays [95% CI: − 6.5, 11.7]. Results for prolonged sitting time were very similar, albeit slightly smaller, to those seen for total sitting time (Table 1). The intervention effects observed overall, and on workdays, occurred via improvements in the intervention group, with smaller changes or no change within the controls (Additional file 3: Table S4). Neither group showed significant changes during non-work time on work days, and on non-work days. No large or significant changes in sitting, standing or stepping were evident during work in the control group (Additional file 3: Table S4). Sitting time outcomes showed weak clustering, even during work hours (ICC = 0.006) and on work days (ICC = 0.003), ranging from < 0.001 on non-work hours on workdays to 0.018 for non-workdays. By contrast, there was greater clustering in stepping time, ranging from ICC = 0.029 on non-work days to ICC = 0.124 overall.

**Table 1:** Intervention effects from baseline to three-months in sitting and activity outcomes over all timeframes (control *n* = 82; intervention *n* = 114) ^a^

Sitting and activity outcomes	Intervention effects		
	Mean difference (95% CI)	*p*	*ICC*
All days, min/16 h waking day			
Sitting	−78.2 (−98.1, −58.4)	<0.001	0.004
Prolonged sitting	−52.2 (−73.5, −31.0)	<0.001	<0.001
Standing	74.8 (57.0, 92.7)	<0.001	0.004
Stepping	2.4 (−7.2, 12.0)	0.628	0.124
Work days, min/16 h waking day			
Sitting	−117.1 (−141.0, −93.2)	<0.001	0.003
Prolonged sitting	−76.7 (−101.0, −52.3)	<0.001	<0.001
Standing	114.1 (92.1, 136.1)	<0.001	<0.001
Stepping	2.6 (−6.5, 11.7)	0.576	0.113
Work hours (on workdays), min/8 h work time			
Sitting	−109.5 (−130.8, −88.2)	<0.001	0.006
Prolonged sitting	− 75.9 (−94.7, −57.2)	<0.001	<0.001
Standing	108.0 (88.3, 127.7)	<0.001	<0.001
Stepping	1.3 (−2.8, 5.5)	0.529	0.083
Non-work hours (on workdays), min/8 h non-work time			
Sitting	−6.8 (−17.0, 3.4)	0.189	<0.001
Prolonged sitting	−0.5 (−13.5, 12.5)	0.941	<0.001
Standing	4.0 (−3.4, 11.4)	0.290	<0.001
Stepping	2.4 (−4.2, 9.1)	0.475	0.033
Non-work days, min/16 h waking day			
Sitting	0.7 (−29.3, 30.7)	0.964	0.018
Prolonged sitting	−0.1 (−30.3, 30.0)	0.995	<0.001
Standing	−1.5 (−28.8, 25.9)	0.915	0.050
Stepping	−0.5 (−13.4, 12.4)	0.941	0.029

Table presents mean difference with 95% confidence interval (CI), *p*-value and intra-cluster correlation (ICC) from linear mixed models adjusting for cluster via random intercept, baseline value of the outcomes, age, gender, BMI, physical quality of life score (AQoL 8D), number of days worked (Monday–Friday), average work duration (Monday–Friday)

^a^ Includes participants with valid data for all covariates, and sitting and activity data for all timeframes (e.g., work days) at baseline and 3 months.

### Temporal variation in workplace sitting

**Fig. 2:**
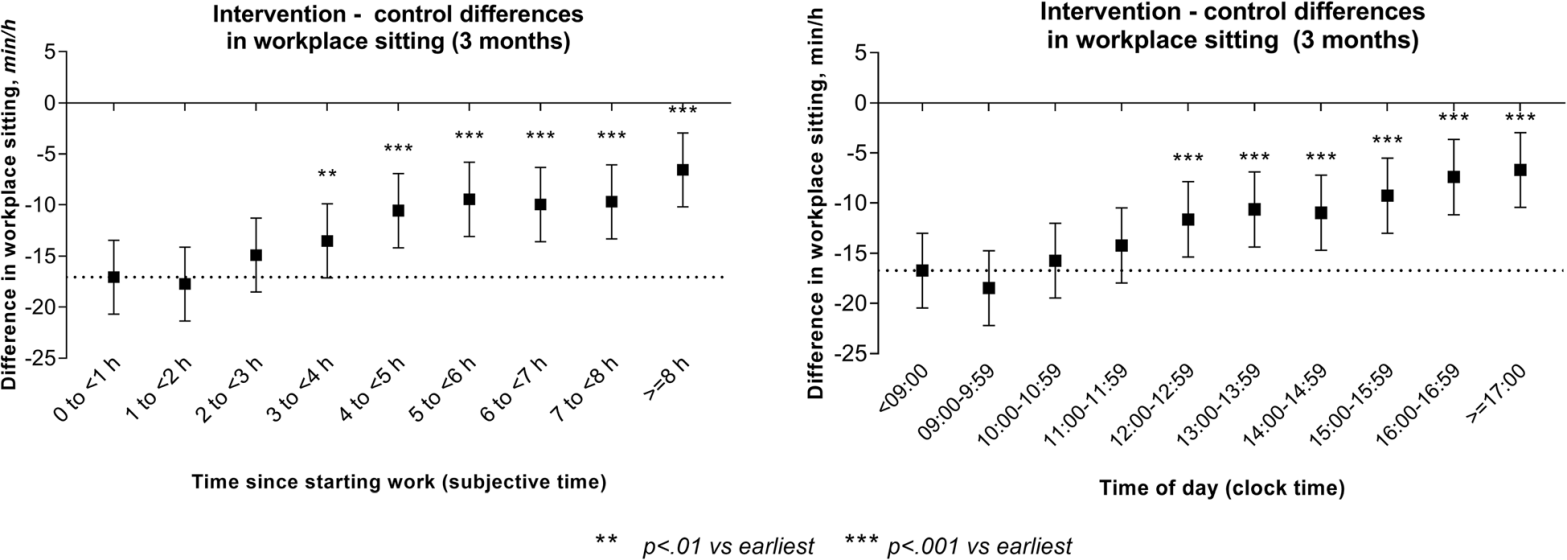
Intervention effects for workplace sitting at three-month follow up by subjective and clock time

At baseline, there was no large nor significant difference between groups at any hour (Additional file 1: Table S5), based on subjective time (overall *p* = 0.281) and clock time (hours of the day; overall *p* = 0.566, respectively). There was also no large nor significant temporal variation in these differences by subjective time (*p for trend* = 0.541) or by clock time (*p for trend* = 0.770). By contrast, there were differences between groups, and temporal variation in these group differences at 3 months, as illustrated in Fig. 2. At 3 months, based on both subjective time and clock time, at each hour of the day, there was a statistically significant difference between groups in favour of the intervention group (all *p* < 0.001; Additional file 3: Table S5). However, these were not equal across the workday by either subjective time or clock time. Relative to the intervention effects seen in the first hour (0 h since starting work; <09:00), effects were significantly less from approximately 4 hours since starting work onwards, and from approximately 12:00 onwards. The strongest group differences in workplace sitting were observed in the second hour (− 17.7 min/h [95% CI: − 21.4, − 14.1]), similarly, between 9 and 10 am (− 18.5 min/h [95% CI: − 22.2, − 14.7]). Conversely, the smallest intervention effect on workplace sitting was seen in the last hour (− 6.6 min/h [95% CI: − 10.2, − 2.9]), or from 5 pm onwards (− 6.7 min/h [95% CI: − 3.0, − 10.4]).

**Fig. 3:**
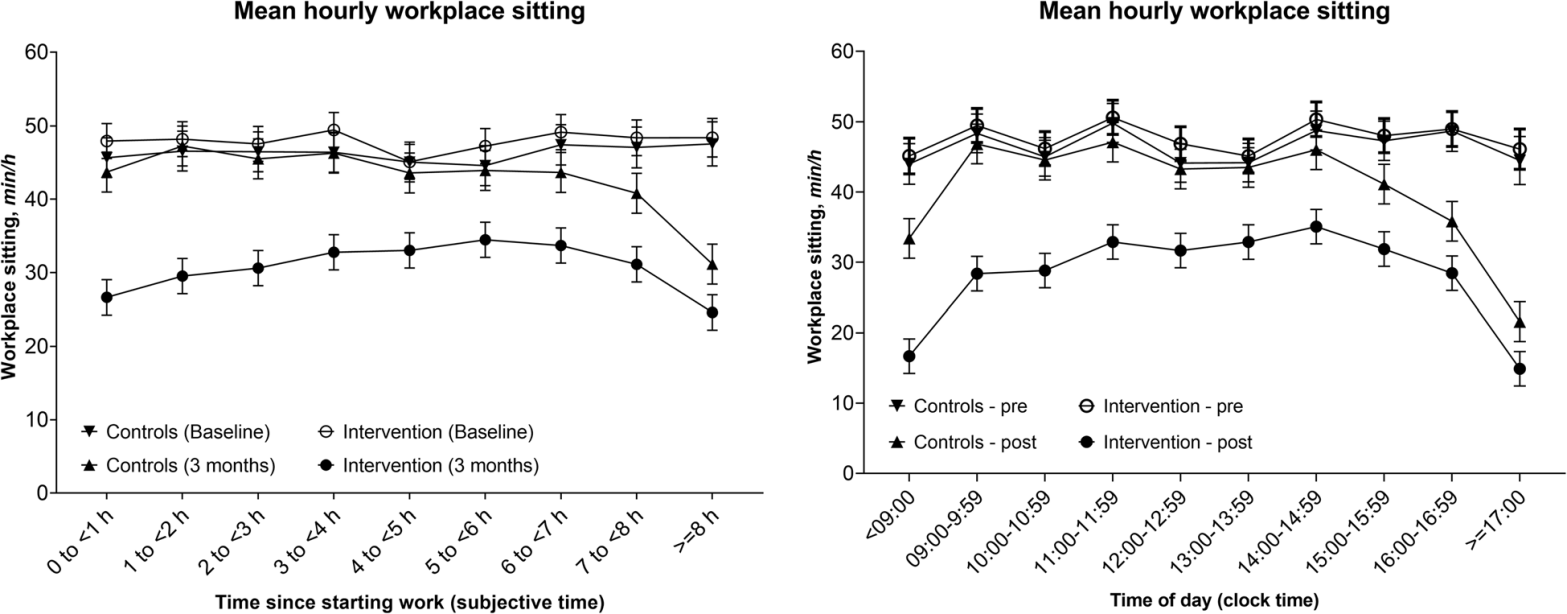
Hourly changes in workplace sitting from baseline to three-months by subjective and clock time

The hourly variation in workplace sitting in each group, before and after intervention (baseline-3 months) is shown in Fig. 3, with further detail in (Additional file 3: Table S6). There had been some degree of variation in hourly sitting at baseline (more by clock time than by subjective time), however, the variations were more pronounced at 3 months in both intervention and control groups.

### Relationship between activity during and outside of work hours

**Fig. 4:**
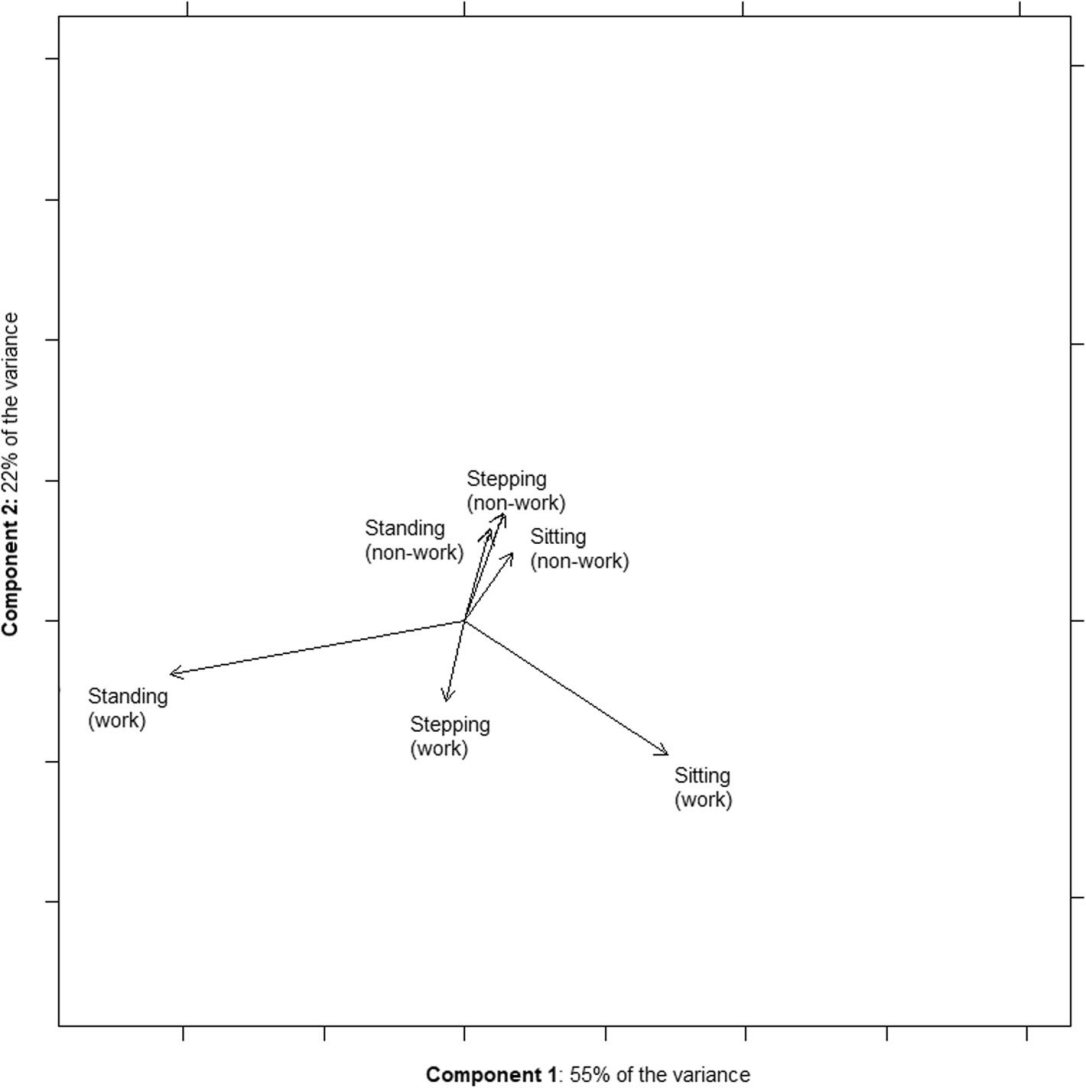
Intervention group bi-plot visualising the relationships between changes in components of daily sitting and activity

**Fig. 5:**
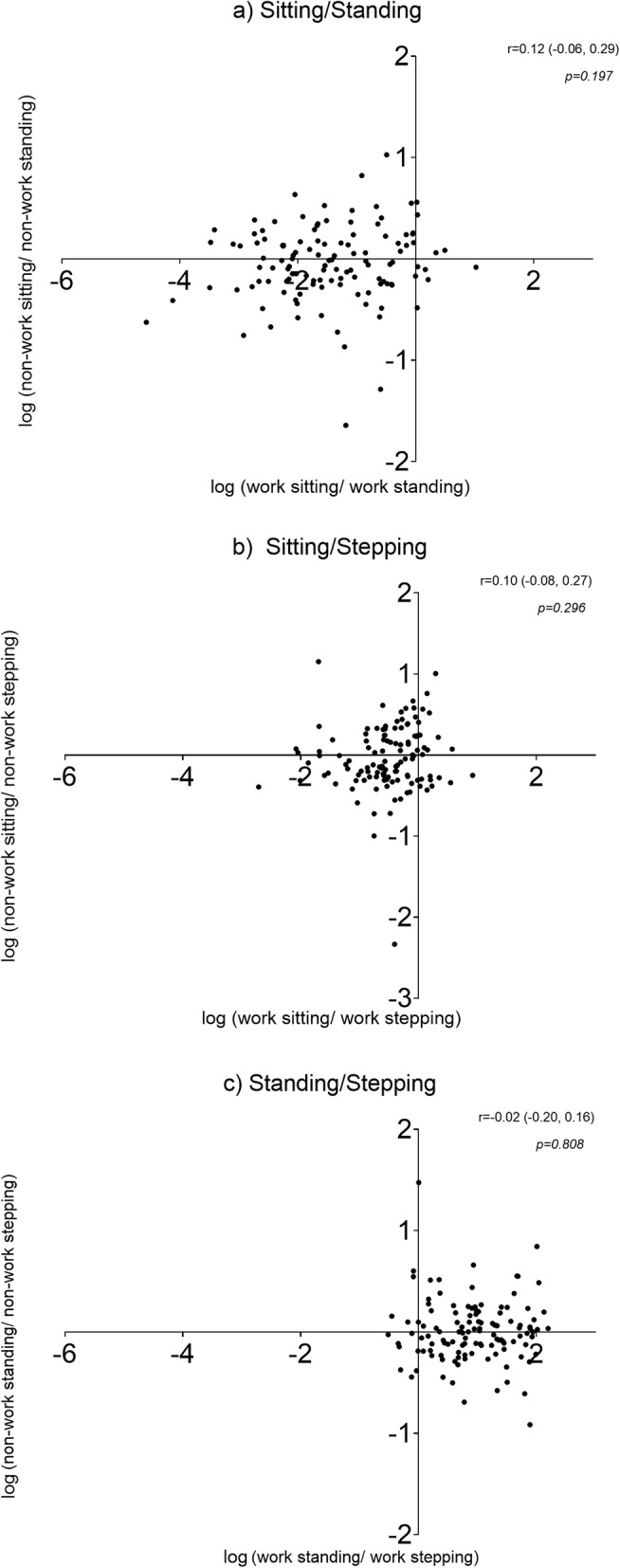
Intervention group log-ratio scatterplots for relationships between activity changes at work and outside of work

The relationships between the intervention group’s changes in the various components of the workday are shown in Fig. 4. The two main components, displayed on the bi-plot, collectively explained 77% of the total variance in the workday changes. The changes in non-work activities (sitting, standing, and stepping) were all grouped together with short links (i.e., they were highly proportional to each other) and appeared largely orthogonal to the changes in work activity. Correspondingly, any correlation between changes in work and non-work activities was only weak (r = − 0.07). Log-ratio scatterplots (Fig. 5) also did not indicate any relationship between activity changes at work and outside of work in terms of: sitting/standing ratios; sitting/stepping ratios; and standing/stepping ratios. Similarly, in the control group, there was no evidence of relationships between changes at work and changes outside of work (r = − 0.09, Additional file 4: Figure S1). There was little change in the correlation in either group when including sleep and non-wear time.

## Discussion

Our detailed examination of changes in sitting, standing, and stepping associated with a workplace-delivered sitting-reduction intervention (Stand Up Victoria) identified effects (favouring intervention) on sitting and standing time, almost exclusively within the intervention delivery setting (the desk-based workplace). There were no large or significant intervention effects on sitting, standing, or stepping outside of work. This finding, along with the lack of any discernible relationship between changes at and outside of work did not support that either compensation or generalisation of behaviour changes had occurred. For sitting time, the overall intervention effect (− 78.2 min/16 h) was the equivalent of 71% of the size of work day effects (− 117.1 min/16 h), and 67% of the size of the work hour effects (− 109.5 min/8 h), which appears to be broadly consistent with participant’s average exposure to the work setting (i.e., work 4.4 days/week, 62% of days). As such, the findings are most consistent with a dilution effect, whereby effects occur exclusively, or nearly exclusively, in the primary intervention setting, and the amount of exposure to that setting (here, the number of days worked per week) may alter the degree of effect that is observed overall.

Previously, findings from the Stand Up Comcare pilot study had shown that the greatest intervention effects on workplace sitting occurred early in the day (i.e., 8–9 am) with noticeably lesser effects around 12–1 pm (typical lunch period), but without a large or clear trend of diminishing effects towards the end of the day [7]. The present trial likewise indicated the greatest intervention effects were observed early in the day, but unlike the pilot study, did not see any specific effects around midday, and saw a clear trend of diminishing effects over time (both by subjective time and clock time). The diminished effects, evident towards the end of the day, may be related to workers experiencing fatigue or muscle discomfort, which is consistent with qualitative research identifying a preference for standing in the morning, and a decline in standing as the day progressed [14]. This timing may also be related to when tasks are undertaken (for example, standing when checking emails at the start of the day). Within the qualitative study, many workers also reported factors relating to comfort or fatigue as contributing to their sitting later in the working day [14]. There is also preliminary evidence that the work environment may impact on breaks in sitting time [26]. More research, for example, across different types of interventions and work environments, and diverse occupations (including shift workers), is needed to further understand the impact of time of day and time since starting work (and contributing factors such as fatigue or discomfort) on behaviour change following intervention.

One workplace intervention involving sit-stand workstations that explored compensation [10] did observe an increase in non-work sitting (+ 8% of non-work time) alongside reductions in work (− 20% of work hours). However, it was not clear whether this reflected compensation, with large changes in the amount of observed time at work (+ 110 min) and outside of work (− 103 min) [10]. Other possible explanations include that working longer hours (by nearly 2 h/day) decreased the available time for physical activity and skewed non-work time proportionally to more sitting. One longitudinal study found that, relative to workers whose occupational activity did not change, those who switched from sedentary to active occupations were more likely to report decreased leisure physical activity [27]. Conversely, those who changed from active to sedentary occupations were more likely to report increased leisure time physical activity [27]. It is important to note, however, that these occupational activity changes were more pronounced than what typically occurs in sitting-reduction interventions such as Stand Up Victoria, which target a change in sitting and active behaviours within the same occupation and environmental setting. We saw no evidence for compensation in Stand Up Victoria, while the evidence more generally for the ‘ActivityStat’ hypothesis is mixed [11, 28]. It is possible that the comparatively small changes of swapping workplace sitting with workplace standing are not sufficiently intense to trigger noticeable compensatory responses, though perhaps enough to limit the extent of change later in the day relative to the morning. The habitual and context-specific nature of sedentary behaviours has been described, with domains such as the desk-based workplace a key setting in which these behaviours habitually occur [29]. A recent study investigating how sitting is perceived by office workers demonstrated that workers often describe the activity which is being undertaken while sitting, rather than the act of sitting itself, suggesting that sitting may often be unlikely to be consciously motivated [30]. As such, this may have implications for whether compensation or generalisation are likely to occur outside of the domain in which the intervention is being delivered. Different types of interventions, that promote different active behaviours and amounts of behaviour change, should be compared to better understand situations in which phenomena like compensation may or may not occur.

There is an emerging body of literature, largely observational, that has noted temporal variations in active and sedentary behaviours, varying throughout the week [31, 32] and across the day [33, 34], with some correlating temporal variations with health outcomes [35]. This study is one of the few to ‘unpack’ when the intervention-generated changes occurred within the primary intervention setting (here, the workplace) and explore how this behaviour change is related to activity occurring in other settings (i.e., outside the workplace), with a view to observing key phenomena: generalisation, compensation and dilution. Key limitations to note are that this study was not powered a priori for these analyses, and the CoDA analyses were exploratory and naïve, ignoring clustering and potential confounding. In general, the effectiveness of the intervention on sitting and activity should be considered from the intention to treat results previously reported [9] in preference to the complete-case results reported here for purposes of comparing domains. Further, changes were based on two seven-day assessments with no observed activity data in-between the time points. Firm conclusions, therefore, cannot be drawn regarding any behaviour changes or temporal patterns that may have been present during the unobserved period and later altered to the present state at the end of intervention.

## Conclusions

In conclusion, these findings indicated the workplace-delivered sitting-reduction Stand Up Victoria trial was behaviourally successful solely within the primary intervention setting (i.e., the workplace). Minimal change, if any, occurred in activity outside of work in response to the intervention, or in relation to changes in activity and sitting behaviour at work, though it is possible fatigue may have limited the extent of improvement later versus earlier in the day while at work. It remains a challenge for workplace-delivered interventions to determine how to promote consistency of behavioural improvement throughout the entire day at work, and what further intervention components, such as wearables, active travel, and home-environmental modifications, may help successfully intervene in domains outside the primary workplace setting (e.g., home, transport and leisure).

## Supplementary information

**Additional file 1: Table S1.** CONSORT 2010 checklist of information to include when reporting a cluster randomised trial. Description: Populated CONSORT Extension for Cluster-Randomised Trials 2012 Checklist.

**Additional file 2:** The TIDieR (Template for Intervention Description and Replication) ChecklistDescription: Populated TIDieR Checklist.

**Additional file 3: Table S1.** Additional data extraction details specific to this study. **Table S2**. Baseline characteristics of participants included (*n* = 196) and excluded (*n* = 35) in this study. **Table S3.** Compliance and amount of work at baseline and 3 months within intervention (*n* = 114) and control participants (*n* = 81). **Table S4.** Adjusted mean changes in sitting and activity outcomes within control (n = 81) and intervention (n = 114) groups^a^. **Table S5.** Between group differences (intervention - control) in workplace sitting time by subjective time (hours since starting work) and clock time. **Table S6.** Variation over time in hourly sitting among the intervention (*n* = 82) and control groups (n = 114) at baseline and 3-month follow up. Description: Supplemental tables presenting additional data relevant to this study.

**Additional file 4. Figure S1.** Control group covariance bi-plot visualising the relationships between changes in components of daily sitting and activity during work and non-work hours. Description: Figure presenting covariance bi-plot for control group to demonstrate the relationships between sitting and active behaviours during and outside of the workplace on work days.

## Data Availability

The availability of the data from the Stand Up Victoria study is subject to the approval of a formal application made to the Chief Investigators.
