# Correction to: Temporal features of sitting, standing and stepping changes in a cluster-randomised controlled trial of a workplace sitting-reduction intervention

**DOI:** 10.1186/s12966-024-01611-9

**Published:** 2024-06-24

**Authors:** Samantha K. Stephens, Elisabeth A. H. Winkler, Elizabeth G. Eakin, Bronwyn K. Clark, Neville Owen, Marj Moodie, Anthony D. La Montagne, David W. Dunstan, Genevieve N. Healy

**Affiliations:** 1 School of Public Health, The University of Queensland, Herston Road, Herston, Brisbane, QLD 4006 Australia; 2 Baker Heart & Diabetes Institute, Melbourne, Australia; 3 Centre for Urban Transitions, Swinburne University of Technology, Melbourne, Australia; 4 Deakin Health Economics, Institute for Health Transformation, Deakin University, Geelong, Australia; 5 Work, Health & Wellbeing Unit, Centre for Population Health Research, Deakin University, Geelong, Australia; 6 Mary MacKillop Institute for Health Research, Australian Catholic University, Brisbane, Australia; 7 School of Sport Science, Exercise & Health, University of Western Australia, Perth, Australia; 8 School of Exercise and Nutrition Sciences, Deakin University, Melbourne, Australia; 9 School of Public Health & Preventive Medicine, Monash University, Melbourne, Australia; 10 School of Physiotherapy, Faculty of Health Sciences, Curtin University, Perth, Australia

10.1186/s12966-019-0879-1

Following publication of the original article [1], it was brought to the journal’s attention that there was an error in ‘Additional file 3: Table S1’. Specifically, in ‘Supplemental Table 4’, the headings of the second and third columns, ‘Intervention (n = 114)’ and ‘Controls (n = 82)’, had been erroneously swapped. The file has since been corrected. This correction does not alter the values detailed in the table.

The authors thank you for reading this erratum and apologize for any inconvenience caused.
