# Supplementary material for: Temporal features of sitting, standing and stepping changes in a cluster-randomised controlled trial of a workplace sitting-reduction intervention
Source: Int J Behav Nutr Phys Act. 2019 Nov 21;16:111. doi: 10.1186/s12966-019-0879-1 (PMC6873403; doi:10.1186/s12966-019-0879-1)
Supplement: Supplementary file 3 — Additional file 3: Table S1. Additional data extraction details specific to this study. Table S2. Baseline characteristics of participants included (n = 196) and excluded (n = 35) in this study. Table S3. Compliance and amount of work at baseline and 3 months within intervention (n = 114) and control participants (n = 81). Table S4. Adjusted mean changes in sitting and activity outcomes within control (n = 81) and intervention (n = 114) groupsa. Table S5. Between group differences (intervention - control) in workplace sitting time by subjective time (hours since starting work) and clock time. Table S6. Variation over time in hourly sitting among the intervention (n = 82) and control groups (n = 114) at baseline and 3-month follow up. Description: Supplemental tables presenting additional data relevant to this study. [file 12966_2019_879_MOESM3_ESM.docx]

**Supplemental Table 1. Additional data extraction details specific to this study.^a^**

| **Timeframe** | **Definitions** | **Summary method** |
| --- | --- | --- |
| Overall (i.e. work time and non-work time on all days) | Valid days required wear for ≥80% of work hours, and ≥10 hours waking wear time (when waking hours were inferred from movement). | Total per day, averaged on valid days, standardised a to 16-hour waking day. |
| Non-work days | Valid day (as above), participant indicated no work at any location. |  |
| Work days | Valid day (as above), participant reported working at the study workplace. |  |
| Work time on work days | Time at the workplace on workdays. For valid work hours data, the monitor needed to be worn for ≥80% of work hours and for a minimum of 1 hour. | Total per day, averaged on valid days, standardised to 8-hours per day (i.e., 16 hours awake, with 8 hours at work and 8 hours non-work time). |
| Non-work time on work days | Time on work days (as above), excluding any times participant reported working (from any location) or workdays on which work times were not specified. |  |
| Work hours, by hour | Hours were defined in two ways: by clock time (i.e., hours of the day), and as subjective time (i.e., number of hours since starting work).  Days were included when valid for work hours data (as above). Hours were included as valid with >30 min of wear time. | For each day separately, during time at work, totals were calculated for each hour, with sparsely populated hours grouped together. These were <9 am, ≥5 pm, and ≥8 hours since starting work. Data were standardised to 60 min/h. |

^a^ General rules used were applied. These are published elsewhere and described in detail (Healy et al., 2016).

**REFERENCES**

Healy, G. N., Eakin, E. G., Owen, N., Lamontagne, A. D., Moodie, M., Winkler, E. A., Fjeldsoe, B. S., Wiesner, G., Willenberg, L. & Dunstan, D. W. 2016. A Cluster Randomized Controlled Trial to Reduce Office Workers' Sitting Time: Effect on Activity Outcomes. *Med Sci Sports Exerc,* 48**,** 1787-97.

**Supplemental Table 2. Baseline characteristics of participants included (n=196) and excluded (n=35) in this study.**

| **Baseline characteristics** | **Intervention (n=114)** | **Control (n=82)** | **All included (n=196)** | **All excluded (n=35)** | ***p*** |
| --- | --- | --- | --- | --- | --- |
| **Age, *years*** | 44.9 (8.9) | 46.0 (9.7) | 45.4 (9.3) | 46.8 (10.2) | 0.426 |
| **Female, n** | 74.0 (65) | 59.0 (72) | 133.0 (68) | 24.5 (71) | 0.631 |
| **1.0 full time equivalent** | 80.7 (92) | 80.5 (66) | 80.6 (158) | 70.0 (25) | 0.128 |
| **BMI, *kg/m^2^*** | 28.7 (6.7) | 28.6 (5.1) | 28.6 (6.0) | 28.4 (6.1) | 0.689 |
| **Workplace sitting, *min/8-h*** | 384.8 (45.4) | 372.1 (40.9) | 379.5 (44.0) | 371.4 (53.8) | 0.311 |
| **Workplace standing, *min/8-h*** | 64.3 (40.0) | 72.1 (32.7) | 67.5 (37.3 | 75.9 (49.9) | 0.291 |
| **Workplace stepping, *min/8-h*** | 30.9 (13.6) | 35.8 (14.0) | 33.0 (14.0) | 32.7 (13.8) | 0.873 |
| **Overall sitting, *min/16-h*** | 625.6 (87.3) | 610.6 (62.9) | 619.4 (78.4) | 628.1 (92.9) | 0.679 |
| **Overall standing, *min/16-h*** | 233.2 (72.0) | 241.6 (47.9) | 236.7 (63.1) | 234.3 (76.3) | 0.787 |
| **Overall stepping, *min/16-h*** | 101.1 (30.7) | 107.7 (24.5) | 103.9 (28.4) | 100.5 (34.6) | 0.552 |

Table presents n (%) or mean ± SD (corrected for clustering using STATA ‘survey commands’). *p* for difference between groups obtained using logistic regression, with random intercept for cluster.

**Supplemental Table 3:** Compliance and amount of work at baseline and 3 months within intervention (n=114) and control participants (n=82)

| **Monitored time** | **Time** | **Mean ± SD or Mean change (95% CI)** | | |
| --- | --- | --- | --- | --- |
|  |  | **Control (n=82)** | **Intervention (n=114)** | ***p*** |
| Valid days, n | Baseline | 6.85 ± 0.47 | 6.92 ± 0.5 | 0.339 |
|  | 3 months | 6.79 ± 0.87 | 6.86 ± 0.73 | 0.612 |
|  | 3 months - Baseline | -0.05 (-0.27, 0.17), p=0.653 | -0.07 (-0.25, 0.12), p=0.492 | 0.915 |
| Valid work days, n | Baseline | 4.22 ± 0.91 | 4.41 ± 0.86 | 0.130 |
|  | 3 months | 4.22 ± 0.98 | 4.2 ± 0.93 | 0.850 |
|  | 3 months - Baseline | 0.00 (-0.24, 0.24), p>.999 | -0.21 (-0.42, -0.01), p=0.044 | 0.193 |
| Valid non-work days, n | Baseline | 2.63 ± 0.82 | 2.49 ± 0.82 | 0.230 |
|  | 3 months | 2.54 ± 0.98 | 2.66 ± 0.87 | 0.596 |
|  | 3 months - Baseline | -0.10 (-0.33, 0.13), p=0.414 | 0.16 (-0.04, 0.36), p=0.115 | 0.100 |
| Wear time: waking hours, h/day | Baseline | 15.73 ± 0.89 | 15.82 ± 0.87 | 0.505 |
|  | 3 months | 15.73 ± 0.90 | 15.65 ± 0.98 | 0.587 |
|  | 3 months - Baseline | -0.05 (-0.31, 0.21), p=0.705 | -0.16 (-0.40, 0.07), p=0.169 | 0.526 |
| Wear time: work day work hours, h/day | Baseline | 8.51 ± 0.96 | 8.46 ± 0.91 | 0.680 |
|  | 3 months | 8.38 ± 1.07 | 8.26 ± 1.02 | 0.680 |
|  | 3 months - Baseline | -0.14 (-0.48, 0.19), p=0.399 | -0.19 (-0.50, 0.11), p=0.212 | 0.827 |
| Wear time: work day non-work hours, h/day | Baseline | 7.74 ± 1.23 | 8.00 ± 1.21 | 0.143 |
|  | 3 months | 7.70 ± 1.32 | 7.93 ± 1.34 | 0.643 |
|  | 3 months - Baseline | -0.04 (-0.45, 0.37), p=0.854 | -0.08 (-0.45, 0.29), p=0.669 | 0.883 |

Table presents mean ± SD (corrected for clustering using STATA ‘survey commands’), with changes over time and differences between groups calculated from mixed models, with a random intercept for cluster.

**Supplemental Table 4.** Adjusted mean changes in sitting and activity outcomes within control (n=82) and intervention (n=114) groups.^a^

| **Sitting and activity outcomes** | **Controls (n=82)** | | **Intervention (n=114)** | |
| --- | --- | --- | --- | --- |
|  | **Mean change (95% CI)** | ***p*** | **Mean change (95% CI)** | ***p*** |
| **All days, min/16h day** | | | | |
| Sitting | **-80.7 (-93.4, -68.0)** | **<0.001** | -2.5 (-17.5, 12.5) | 0.747 |
| Prolonged sitting | **-63.0 (-76.5, -49.5)** | **<0.001** | -10.7 (-26.8, 5.3) | 0.189 |
| Standing | **77.3 (65.8, 88.7)** | **<0.001** | 2.4 (-11.1, 16.0) | 0.723 |
| Stepping | 2.7 (-3.8, 9.2) | 0.416 | 0.3 (-6.8, 7.4) | 0.932 |
| **Work days, min/16h day** | | | | |
| Sitting | **-125.2 (-140.5, -109.9)** | **<0.001** | -8.2 (-26.2, 9.9) | 0.376 |
| Prolonged sitting | **-97.7 (-113.2, -82.2)** | **<0.001** | **-21.0 (-39.4, -2.6)** | **0.025** |
| Standing | **121.9 (107.8, 136.0)** | **<0.001** | 7.9 (-8.8, 24.5) | 0.356 |
| Stepping | 3.2 (-2.9, 9.3) | 0.310 | 0.6 (-6.1, 7.3) | 0.867 |
| **Work hours, min/8h work time** | | | | |
| Sitting | **-117.5 (-131.1, -103.8)** | **<0.001** | -7.9 (-24.0, 8.1) | 0.333 |
| Prolonged sitting | **-90.9 (-102.8, -79.9)** | **<0.001** | **-14.9 (-29.1, -0.8)** | **0.039** |
| Standing | **115.5 (102.9, 128.1)** | **<0.001** | 7.4 (-7.4, 22.3) | 0.327 |
| Stepping | 2.3 (-0.5, 5.0) | 0.106 | 0.9 (-2.1, 4.0) | 0.547 |
| **Non-work hours (on work days), min/8h non-work time** | | | | |
| Sitting | -4.7 (-11.2, 1.9) | 0.161 | 2.2 (-5.6, 9.9) | 0.584 |
| Prolonged sitting | -4.9 (-13.2, 3.4) | 0.251 | -4.4 (-14.2, 5.5) | 0.383 |
| Standing | -2.9 (-1.9, 7.6) | 0.236 | -1.1 (-6.7, 4.5) | 0.694 |
| Stepping | 1.4 (-2.9, 5.7) | 0.530 | 1.0 (-6.0, 3.9) | 0.686 |
| **Non-work days, min/16h day** | | | | |
| Sitting | 6.1 (-13.3, 25.5) | 0.538 | 5.4 (-17.2, 28.0) | 0.639 |
| Prolonged sitting | 4.9 (-14.4, 24.2) | 0.620 | 5.0 (-17.8, 27.8) | 0.668 |
| Standing | -6.0 (-24.0, 12.1) | 0.517 | -4.5 (-24.9, 16.0) | 0.668 |
| Stepping | -0.6 (-9.0, 7.8) | 0.896 | -0.1 (-9.8, 9.6) | 0.988 |

Table presents changes within intervention and control groups with 95% confidence interval (CI) and p values obtained as marginal means from linear mixed models adjusting for cluster via random intercept, and covariates as fixed effects. Adjusted for baseline value of the outcomes, age, sex, body mass index, physical quality of life score (AQoL-8D), number of days worked (Monday-Friday), and average daily work duration (Monday-Friday). Significant results at p<0.001 are indicated in bold.

^a^ Includes participants with valid data for all covariates, and sitting and activity data for all timeframes (e.g., work days) at baseline and 3 months.

**Supplemental Table 5.** Between group differences (intervention - control) in workplace sitting time by subjective time (hours since starting work) and clock time.

| **Hour** | **Baseline** | | | | **Three months** | | | |
| --- | --- | --- | --- | --- | --- | --- | --- | --- |
|  | **Intervention-Control** | | **‘Hour’** | | **Intervention-Control** | | **‘Hour’** | |
|  | **Mean diff (95% CI)** | ***p*** | **Mean diff (95% CI)** | ***p*** | **Mean diff (95% CI)** | ***p*** | **Mean diff (95% CI)** | ***p*** |
| **Subjective hour** |  | *0.281* ^a^ |  | *0.541* ^b^ |  | *<0.001* ^a^ |  | *<0.001* ^b^ |
| 0 to <1 | 2.3 (-1.3, 5.9) | 0.218 | 0 (referent) |  | -17.1 (-20.7, -13.4) | <0.001 | 0 (referent) |  |
| 1 to <2 | 1.6 (-2, 5.2) | 0.378 | -0.6 (-3.3, 2.0) | 0.632 | -17.7 (-21.4, -14.1) | <0.001 | -0.7 (-3.3, 2.0) | 0.622 |
| 2 to <3 | 1.1 (-2.5, 4.7) | 0.557 | -1.2 (-3.8, 1.4) | 0.378 | -14.9 (-18.5, -11.3) | <0.001 | 2.2 (-0.5, 4.8) | 0.112 |
| 3 to <4 | 3 (-0.6, 6.7) | 0.099 | 0.8 (-1.9, 3.4) | 0.564 | -13.5 (-17.1, -9.9) | <0.001 | 3.6 (0.9, 6.2) | 0.009 |
| 4 to <5 | 0 (-3.6, 3.6) | 0.987 | -2.2 (-4.9, 0.4) | 0.096 | -10.6 (-14.2, -6.9) | <0.001 | 6.5 (3.8, 6.2) | <0.001 |
| 5 to <6 | 2.7 (-1, 6.3) | 0.153 | 0.4 (-2.3, 3.0) | 0.777 | -9.5 (-13.1, -5.8) | <0.001 | 7.6 (4.9, 10.3) | <0.001 |
| 6 to <7 | 1.7 (-1.9, 5.4) | 0.357 | -0.6 (-3.2, 2.1) | 0.686 | -10 (-13.6, -6.3) | <0.001 | 7.1 (4.4, 9.8) | <0.001 |
| 7 to <8 | 1.3 (-2.4, 5) | 0.486 | -1.0 (-3.7, 1.8) | 0.495 | -9.7 (-13.3, -6.1) | <0.001 | 7.4 (4.7, 10.1) | <0.001 |
| ≥8 | 0.8 (-3.1, 4.8) | 0.678 | -1.4 (-4.5, 1.7) | 0.372 | -6.6 (-10.2, -2.9) | <0.001 | 10.5 (7.8, 13.2) | <0.001 |
| **Clock hour** |  | *0.566* ^a^ |  | *0.770* ^b^ |  | *<0.001* ^a^ |  | <0.001 ^b^ |
| <09:00 | 1.1 (-2.7, 5) | 0.566 | 0 (referent) | - | -16.7 (-20.5, -13.0) | <0.001 | 0 (referent) | - |
| 09:00 to 09:59 | 1.1 (-2.5, 4.8) | 0.548 | -0.0 (-3.1, 3.1) | .997 | -18.5 (-22.2, -14.7) | <0.001 | -1.7 (-4.7, 1.2) | 0.248 |
| 10:00 to 10:59 | 1.2 (-2.5, 4.8) | 0.529 | 0.0 (-3.0, 3.1) | .977 | -15.7 (-19.5, -12.0) | <0.001 | 1.0 (-2.0, 4.0) | 0.514 |
| 11:00 to 11:59 | 0.8 (-2.9, 4.5) | 0.669 | -0.3 (-3.4, 2.7) | .832 | -14.2 (-18, -10.5) | <0.001 | 2.5 (-0.5, 5.5) | 0.098 |
| 12:00 to 12:59 | 2.8 (-0.9, 6.4) | 0.140 | 1.6 (-1.4, 4.7) | .299 | -11.6 (-15.4, -7.9) | <0.001 | 5.1 (2.1, 8.1) | <0.001 |
| 13:00 to 13:59 | 0.9 (-2.7, 4.6) | 0.614 | -0.2 (-3.3, 2.9) | .905 | -10.6 (-14.4, -6.9) | <0.001 | 6.1 (3.1, 9.1) | <0.001 |
| 14:00 to 14:59 | 1.6 (-2.1, 5.3) | 0.401 | 0.4 (-2.6, 3.5) | .777 | -11.0 (-14.7, -7.2) | <0.001 | 5.8 (-2.8, 8.7) | <0.001 |
| 15:00 to 15:59 | 0.7 (-3, 4.5) | 0.694 | -0.4 (-3.5, 2.7) | .809 | -9.3 (-13.0, -5.5) | <0.001 | 7.5 (4.5, 10.4) | <0.001 |
| 16:00 to 16:59 | 0.3 (-3.5, 4.1) | 0.882 | -0.8 (-4.1, 2.4) | .611 | -7.4 (-11.1, -3.6) | <0.001 | 9.3 (6.4, 12.3) | <0.001 |
| ≥17:00 | 1.6 (-2.8, 6.1) | 0.478 | 0.5 (-3.5, 4.5) | .816 | -6.7 (-10.4, -3.0) | 0.001 | 10.0 (7.0, 13.0) | <0.001 |

^a^ Overall between group difference averaged across hours

^b^ *p* for trend in hourly variation in intervention – control differences

**Supplemental Table 6.** Variation over time in hourly sitting among the intervention (n=82) and control groups (n=114) at baseline and 3-month follow up.

| **Hour** | **Adjusted mean (95% CI) sitting time per hour, *min/h*** ^a^ | | | | | | | |
| --- | --- | --- | --- | --- | --- | --- | --- | --- |
|  | **Controls** | | | | **Intervention** | | | |
|  | **Baseline** | | **Three months** | | **Baseline** | | **Three months** |  |
|  | **Mean (95% CI)** | ***p*** | **Mean (95% CI)** | ***p*** | **Mean (95% CI)** | ***p*** | **Mean (95% CI)** | ***p*** |
| **Subjective time** |  | *0.233 ^b^* |  | <0.001 *^b^* |  | 0.621 *^b^* |  | 0.342 *^b^* |
| 0 to <1 h | 45.7 (43.0, 48.4) | - | 43.7 (41.0, 46.4) | - | 47.9 (45.6, 50.3) |  | 26.6 (24.2, 29.0) | <0.001 |
| 1 to <2 h | 46.6 (43.9, 49.3) | 0.389 | 47.3 (44.5, 50.0) | 0.001 | 48.2 (45.8, 50.6) | 0.762 | 29.5 (27.1, 31.9) | <0.001 |
| 2 to <3 h | 46.5 (43.8, 49.2) | 0.440 | 45.5 (42.8, 48.2) | 0.086 | 47.6 (45.2, 49.9) | 0.654 | 30.6 (28.2, 33.0) | <0.001 |
| 3 to <4 h | 46.4 (43.7, 49.1) | 0.477 | 46.3 (43.6, 49.0) | 0.014 | 49.5 (47.1, 51.8) | 0.074 | 32.8 (30.4, 35.2) | <0.001 |
| 4 to <5 h | 45.1 (42.4, 47.8) | 0.568 | 43.6 (40.9, 46.3) | 0.916 | 45.1 (42.7, 47.5) | 0.001 | 33.0 (30.6, 35.4) | <0.001 |
| 5 to <6 h | 44.6 (41.9, 47.3) | 0.306 | 43.9 (41.2, 46.6) | 0.833 | 47.2 (44.8, 49.6) | 0.418 | 34.5 (32.1, 36.9) | <0.001 |
| 6 to <7 h | 47.4 (44.7, 50.2) | 0.098 | 43.7 (40.9, 46.4) | 0.968 | 49.1 (46.7, 51.5) | 0.160 | 33.7 (31.3, 36.1) | <0.001 |
| 7 to <8 h | 47.1 (44.3, 49.9) | 0.196 | 40.8 (38.1, 43.5) | 0.006 | 48.4 (46.0, 50.8) | 0.610 | 31.1 (28.7, 33.5) | <0.001 |
| ≥8 h | 47.6 (44.5, 50.6) | 0.125 | 31.2 (28.4, 33.9) | <.001 | 48.4 (45.8, 51.0) | 0.650 | 24.6 (22.2, 27.0) | 0.019 |
| **Clock time** |  | *0.183 ^b^* |  | *<0.001 ^b^* |  | *<0.001 ^b^* |  | *<0.001 ^b^* |
| <09:00 | 44.0 (41.1, 46.9) | - | 33.4 (30.6, 36.2) | - | 45.1 (42.6, 47.7) | - | 16.7 (14.2, 19.1) | - |
| 09:00 to 09:59 | 48.4 (45.6, 51.1) | 0.001 | 46.9 (44, 49.7) | <0.001 | 49.5 (47.1, 51.9) | <0.001 | 28.4 (25.9, 30.8) | <0.001 |
| 10:00 to 10:59 | 45.0 (42.3, 47.8) | 0.403 | 44.6 (41.7, 47.4) | <0.001 | 46.2 (43.8, 48.6) | 0.289 | 28.8 (26.4, 31.3) | <0.001 |
| 11:00 to 11:59 | 49.8 (47.1, 52.6) | <0.001 | 47.1 (44.3, 49.9) | <0.001 | 50.6 (48.2, 53.0) | <0.001 | 32.9 (30.4, 35.3) | <0.001 |
| 12:00 to 12:59 | 44.1 (41.4, 46.9) | 0.916 | 43.3 (40.5, 46.1) | <0.001 | 46.9 (44.5, 49.3) | 0.078 | 31.7 (29.2, 34.1) | <0.001 |
| 13:00 to 13:59 | 44.2 (41.4, 46.9) | 0.879 | 43.5 (40.7, 46.3) | <0.001 | 45.1 (42.7, 47.6) | 0.997 | 32.9 (30.4, 35.3) | <0.001 |
| 14:00 to 14:59 | 48.8 (46.0, 51.5) | <0.001 | 46.0 (43.2, 48.9) | <0.001 | 50.3 (47.9, 52.8) | <0.001 | 35.1 (32.6, 37.5) | <0.001 |
| 15:00 to 15:59 | 47.3 (44.5, 50.1) | 0.008 | 41.1 (38.3, 44) | <0.001 | 48 (45.6, 50.5) | 0.004 | 31.9 (29.4, 34.3) | <0.001 |
| 16:00 to 16:59 | 48.7 (45.8, 51.6) | 0.001 | 35.8 (33, 38.7) | 0.038 | 49 (46.5, 51.5) | <0.001 | 28.5 (26.0, 30.9) | <0.001 |
| ≥ 17:00 | 44.5 (41.1, 47.9) | 0.759 | 21.6 (18.8, 24.4) | <0.001 | 46.1 (43.3, 49.0) | 0.443 | 14.9 (12.4, 17.3) | 0.061 |

Table presents adjusted mean and 95% CI from linear mixed models. ^a^ adjusted for baseline value of the outcomes, age, sex, body mass index, physical quality of life score (AQoL-8D), number of days worked (Mon-Fri), and average daily work duration (Monday-Friday workdays). Includes participants with valid data for all covariates and baseline and 3 month data on work activity. ^b^ p for trend.
