# Supplementary figures and images for: Temporal features of sitting, standing and stepping changes in a cluster-randomised controlled trial of a workplace sitting-reduction intervention
Source: Int J Behav Nutr Phys Act. 2019 Nov 21;16:111. doi: 10.1186/s12966-019-0879-1 (PMC6873403; doi:10.1186/s12966-019-0879-1)

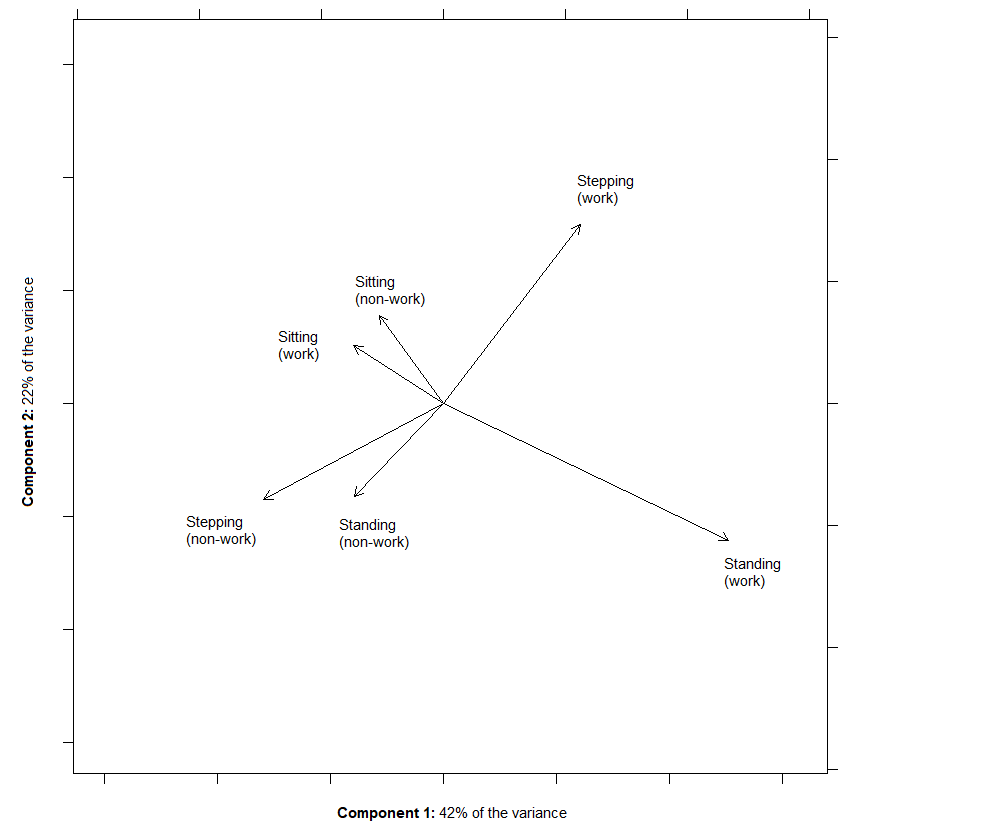

Supplement: Supplementary file 4 — Additional file 4. Figure S1. Control group covariance bi-plot visualising the relationships between changes in components of daily sitting and activity during work and non-work hours. Description: Figure presenting covariance bi-plot for control group to demonstrate the relationships between sitting and active behaviours during and outside of the workplace on work days. [file 12966_2019_879_MOESM4_ESM.tiff]
